# Effects of Breastfeeding on Endometriosis-Related Pain: A Prospective Observational Study

**DOI:** 10.3390/ijerph182010602

**Published:** 2021-10-10

**Authors:** Romana Prosperi Porta, Chiara Sangiuliano, Alessandra Cavalli, Laila Cristine Hirose Marques Pereira, Luisa Masciullo, Ilaria Piacenti, Sara Scaramuzzino, Maria Federica Viscardi, Maria Grazia Porpora

**Affiliations:** 1 Il Melograno, Maternity and Childbirth Empowerment Centres, 00179 Rome, Italy; prosperiporta@gmail.com; 2 Department of Maternal and Child Health and Urological Sciences, Sapienza University of Rome, 00161 Rome, Italy; chiara.sangiuliano@uniroma1.it (C.S.); alessandra.cavalli@uniroma1.it (A.C.); hiroselaila@gmail.com (L.C.H.M.P.); luisa.masciullo@uniroma1.it (L.M.); sara.scaramuzzino@uniroma1.it (S.S.); mariafederica.viscardi@uniroma1.it (M.F.V.); 3 Department of Obstetrics and Gynecology, Santa Maria Della Stella Hospital, 05018 Orvieto, Italy; ilaria_piacenti@libero.it

**Keywords:** endometriosis, breastfeeding, exclusive breastfeeding, acyclic chronic pelvic pain, dysmenorrhea, postpartum amenorrhea

## Abstract

Endometriosis is a gynecological estrogen-dependent disease whose commonest pain symptoms are dysmenorrhea, dyspareunia, and acyclic chronic pelvic pain (CPP). Hormonal changes occurring during breastfeeding seem to reduce pain and disease recurrence. The aim of this observational prospective study was to assess the effect of breastfeeding on pain and endometriotic lesions in patients with endometriosis and to evaluate a possible correlation between the duration of breastfeeding, postpartum amenorrhea, and pain. Out of 156 pregnant women with endometriosis enrolled, 123 who breastfed were included in the study and were monitored for 2 years after delivery; 96/123 exclusively breastfed for at least 1 month. Mode of delivery, type and duration of breastfeeding, intensity of pain symptoms, and lesion size before pregnancy and during the 24-month follow-up were analyzed. All patients experienced a significant reduction in dysmenorrhea proportional to the duration of breastfeeding. CPP was significantly reduced only in women who exclusively breastfed. No significant improvement in dyspareunia was observed. Ovarian endometriomas were significantly reduced. Therefore, breastfeeding, particularly if exclusive, may cause improvement in dysmenorrhea and CPP proportional to the duration of breastfeeding, as well as a reduction in the size of ovarian endometriomas.

## 1. Introduction

Endometriosis is a gynecological, estrogen-dependent, inflammatory disease, defined as the presence of functional endometrial tissue outside the uterine cavity, affecting up to 10% of the general female population [1]. Endometriosis is associated with pelvic pain in 30%–80% of cases [2] and with infertility in 30%–40% of cases [3]. Several mechanisms seem to be involved in the pathogenesis of pain, such as peritoneal inflammation, adhesions, deep infiltration with tissue damage, fibrotic thickening, nerve infiltration, and neoformation [4,5,6,7,8]. There is growing evidence that the hormonal environment plays a leading role in both pathogenesis and control of endometriosis. Increased expression of estrogen receptors (ER) has been found in ectopic implants, suggesting that a reduction in estrogen blood levels might be responsible for regression of the disease [9]. In addition, progesterone resistance has been proposed, as endometriotic lesions show an overall reduction in progesterone receptors (PR) compared to the eutopic endometrium [10,11,12,13]. The altered local hormonal milieu allows for proliferation of endometrial cells and implants as a result of mesothelial overgrowth and/or immune-mediated clearance evasion [14]. Its dependence on sex hormones is also confirmed by the evidence that hormonal therapy can delay surgery, control pain, and prevent recurrence; however, pain often recurs after treatment discontinuation [15]. Pregnancy has a positive effect on endometriosis and its associated pain due to hormonal changes [16], but obstetric complications have been reported in affected patients [17,18,19,20,21,22,23,24].

The hormonal environment seems to have an overwhelming influence on the development of endometriosis, suggesting that some physiological mechanisms, such as breastfeeding, may influence the course of the disease.

In 2020, the WHO published new recommendations on the benefits of breastfeeding with increased emphasis on the advantages it brings to both mother and newborn [25]. According to the WHO, breastfeeding and artificial feeding can be classified as follows:

- Exclusive breastfeeding: the infant only receives breast milk without any additional food or drink, not even water.
- Predominant breastfeeding: maternal milk, maternal milk squeezed or donated, plus non-nutritional liquids such as water, infusions, and sugary drinks.
- Complementary feeding: maternal milk and any other drink or food added, including non-human milk.
- Artificial feeding: no maternal milk feeding, only other sources of drinks and food, including non-human milk.

Type and duration of breastfeeding play crucial roles in the hormonal feedback pathways, influencing the production of gonadotropin-releasing hormone (GnRH) [26]. The mechanism by which breastfeeding may have a protective impact on women with endometriosis involves the effect of prolactin (PRL) on the hypothalamic–pituitary axis. Breastfeeding is directly supported by the action of two hormones, PRL and oxytocin, while estrogens are indirectly involved. After childbirth, the sudden loss of both estrogen and progesterone produced by the placenta stimulates the pituitary release of PRL to provide its lactogenic effect and induce milk production. Mechanical stimulation of the nipple is the most important regulator of PRL secretion, and it causes the pituitary release of gonadotropin-releasing hormone (GnRH), follicle-stimulating hormone (FSH), and luteinizing hormone (LH). PRL in turn inhibits GnRH release and it hinders FSH and LH production. Sucking stimulus triggers oxytocin release, which gives rise to milk ejection. In addition, sucking is responsible for the strong emotional connection between mother and newborn through the skin-to-skin contact [27,28,29]. Prolonged breastfeeding may increase the duration of postpartum amenorrhea and promote circulation of oxytocin [30]. 

However, the increased risk of obstetric complications in women with endometriosis including adverse outcomes of pregnancy and a higher rate of cesarean sections [16,17,18,19,20,21,22,23,30,31,32] may cause a delay in the onset of breastfeeding and thus hinder exclusive breastfeeding in these women [33]. 

Breastfeeding may improve endometriosis and reduce its symptoms considering the role of hormones involved in the pathogenesis of endometriosis. Therefore, it may be presumed that the hormonal feedback mechanism triggered by breastfeeding can negatively affect the development of the disease and reduce recurrence. Some studies suggest that breastfeeding may protect against endometriosis [34,35,36,37,38,39], but its positive impact on the disease has been poorly evaluated and this topic has been little debated in the literature.

The primary objective of this study was to assess the effect of breastfeeding on dysmenorrhea, dyspareunia, acyclic chronic pelvic pain (CPP), endometriotic lesions, and recurrence rate in women with histologically confirmed endometriosis.

The secondary objective was to analyze the relationship between the duration and intensity of breastfeeding and postpartum amenorrhea and pain, as well as endometriosis recurrence.

## 2. Materials and Methods

Between January 2017 and June 2019, we enrolled 156 pregnant women affected by histologically confirmed endometriosis, followed by the outpatient service of Endometriosis and pelvic pain at the Policlinico Umberto I, Sapienza University of Rome. Written informed consent was obtained from all patients. The study was part of a larger research project investigating the link between endometriosis and pregnancy approved by the Institutional Ethics Committee (C.E. n. 4776/17). Inclusion criteria were surgical and histological diagnosis of endometriosis before pregnancy, ongoing pregnancy, and at least one month of breastfeeding. 

Exclusion criteria were exclusive artificial feeding, administration of hormonal contraception after childbirth, and lack of compliance to perform periodical clinical and sonographic follow-up for 24 months after pregnancy. Medical history, age, body mass index (BMI), parity, rASRM stage [40] at surgery performed before pregnancy, clinical features, presence and intensity of pain symptoms, presence of endometriotic lesions, and medical treatment before pregnancy were recorded. 

All breastfeeding women (exclusive, predominant, and complementary) were divided into 6 subgroups according to the duration of breastfeeding: 1–2 months (A), 3–5 months (B), 6–8 months (C), 9–11 months (D), 12–18 months (E), and >19 months (F). Data obtained in patients who exclusively breastfed were also separately analyzed. Intensity of dysmenorrhea, dyspareunia, and CPP was assessed by using a 10-point visual analog scale (VAS) ranging from 0 (no pain) to 10 (unbearable pain). Pain symptoms reported by the patients were evaluated before pregnancy (T0), at 3 (T3), 6 (T6), 12 (T12), and 24 (T24) months after childbirth, either during the consultations or by sending specific pain questionnaires by email. Prevalence and intensity of dysmenorrhea were evaluated after the first resumed menstruation until 24 months after delivery. Mean postpartum amenorrhea was also evaluated. Clinical and transvaginal ultrasound (TVUS) examinations (GE Voluson E6, suprapubic 3.5 MHz volume probe and transvaginal 6 MHz volume probe, with 3D scan, GE Healthcare, Milwaukee, WI, USA) were performed at the beginning of pregnancy, 6 months after delivery, and at the end of the follow-up, in order to detect any change in the size of ovarian endometriomas or deep endometriosis (DIE), if present. 

Statistical analysis was performed using Microsoft Excel and SPSS (version 26 for iMac; IBM, SPSS Statistics, Bologna, Italy) provided by ‘Sapienza’ University of Rome. A preliminary descriptive analysis was performed to obtain average values and standard deviations regarding patients’ general characteristics. The Shapiro–Wilk test was performed to test for a normal distribution. Categorical variables were evaluated using the McNemar Test; continuous variables were evaluated using Student’s paired *t*-test or the Wilcoxon test, as appropriate. The statistical dependence between the rankings of two variables was calculated using Spearman’s rank correlation. Confidence interval was set at 95% and alpha value at 0.05 (*p*-value < 0.05).

## 3. Results

A total of 123 breastfeeding women (exclusive or complementary) were included in the study out of 156 recruited; 33 were excluded: 18 due to exclusive use of artificial feeding and 15 due to having requested hormonal contraception after delivery. Median maternal age at childbirth was 34 years, mean 33.8 ± 4.7 (range 25–38 years). Median BMI was 22, mean 22.2 ± 2.7. All patients had surgical/histological diagnosis of endometriosis: 66 patients were diagnosed with endometriosis stage III (53.6%) and 57 with stage IV (46.4%). At surgery, all patients had ovarian endometriomas; 76 (61.8%) were unilateral and 47 (38.2%) were bilateral; peritoneal lesions were present in 74 patients (60.1%), adhesions in 62 (50.4%), and deep infiltrating endometriosis (DIE) in 39 (31.7%). Before pregnancy, 51 patients (41.4%) received combined estrogen–progestin therapy for contraception and/or as a treatment for endometriosis-related pain, and 109 (88.6%) used non-steroidal anti-inflammatory drugs (NSAIDs) to control pain. At the beginning of pregnancy, TVUS examination did not reveal DIE lesions, whereas ovarian endometriomas were detected in 51 women (41.4%); 12 had bilateral endometriomas (23.5%) and 39 (76.5%) had unilateral endometriomas. The largest diameter of ovarian endometriomas was 5 cm, mean 3.25 cm ± 0.91 cm. Thirty-nine patients (31.7%) had had previous pregnancies. A total of 105 women (85.4%) delivered at term, and 18 (14.6%) had preterm delivery (< 37 weeks of gestational age); 60 women had vaginal delivery (48.8 %), and 63 (51.2%) underwent cesarean section. All the characteristics of the study population are reported in Table 1.

All patients completed the 24-month follow-up. Exclusive breastfeeding was provided by 96 women (range 1–8 months) for at least 1 month, then continued with predominant or complementary breastfeeding, while only 27 provided predominant or complementary breastfeeding from childbirth (range 1 to >19 months).

Median postpartum amenorrhea was 5 months, mean 5.8 ± 4.3 months (range 2–16 months). According to the duration of breastfeeding, 29 patients were included in subgroup A, 33 in subgroup B, 41 in subgroup C, 10 in subgroup D, 6 in subgroup E, and 4 in subgroup F. No correlation was found between gestational age, type of delivery, and type of breastfeeding. The median dysmenorrhea VAS score before pregnancy was 7, mean 6.1 ± 3.6. After menstrual period resumed, all patients experienced reduced intensity of dysmenorrhea during the whole follow-up period, but the difference was statistically significant only in groups C (median VAS score 4, mean 3.8 ± 1.8 vs. T0 median VAS score 5.8, mean 5.2 ± 2.2) and D (median VAS score 2, mean 2.5 ± 1.9 vs. T0 median VAS score 6.5, mean 6.1 ± 2.1). In fact, patients in group C reported significant pain relief during the first 6 months of follow-up (*p* = 0.02), and patients in group D experienced pain improvement in the whole 24-month follow-up period (*p* = 0.04). Before pregnancy, the median CPP VAS score was 4.5, mean 4.8 ± 2. All groups reported improvement in CPP, but it was not statistically significant. Overall, the prevalence of dyspareunia decreased, particularly in group F (60% before pregnancy vs. 40% at T24), but also this reduction was not statistically significant. Moreover, the intake of NSAIDs was reduced from 88.6% before pregnancy to 12.2% during the whole follow-up period. 

Although quality of life (QoL) evaluation was not an aim of our study, 81 patients (65.8%) reported a significant improvement in their health status in terms of self-perception of pain-related QoL. Among them, 60 patients (74%) exclusively breastfed. 

### 3.1. Exclusive Breastfeeding 

Of the 123 patients included in the study, 96 (78%) exclusively breastfed for at least 1 month. Late preterm delivery occurred in 12 women (12.5%), and cesarean section was performed in 45 (46.9%). Median postpartum amenorrhea was 5 months, mean 6.2 ± 4.4 months (range 3–16 months). Patients were divided into groups A, B, and C, according to the duration of exclusive breastfeeding, because no women exclusively breastfed for more than 8 months: 28 patients were included in subgroup A, 31 in subgroup B, and 37 in subgroup C. 

Before pregnancy, median VAS of dysmenorrhea was 6.5, mean 6.1 ± 3.5. All patients presented a reduction in the overall VAS score in the whole follow-up period from the first menstruation after childbirth. Improvement of dysmenorrhea at the 3- and 6-month follow-ups was analyzed only in women with early resumed period, as shown in Table 2. 

**Figure 1:**
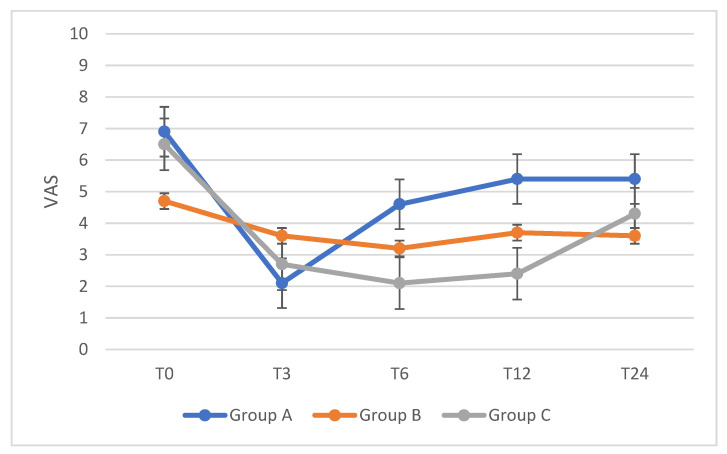
Changes of dysmenorrhea VAS score over time in exclusively breastfeeding women compared to the months of breastfeeding. T0 = before pregnancy; T3 = 3-month follow-up; T6 = 6-month follow-up; T12 = 12-month follow-up; T24 = 24-month follow-up. A = 1–2 months of breastfeeding; B = 3–5 months of breastfeeding; C = 6–8 months of breastfeeding.

In particular, patients of group A experienced a significant pain improvement during the first 3 months (median VAS 2, *p* = 0.01), patients in group B felt pain relief for 6 months (median VAS 3.2, *p* = 0.002), and patients in group C reported pain improvement throughout the whole follow-up period (median VAS 4.3 at T24, *p* < 0.001). Pain intensity increased over the time during the follow-up, but it never reached the value reported before pregnancy. Changes over time in dysmenorrhea compared to duration of breastfeeding are shown in Figure 1.

**Figure 2:**
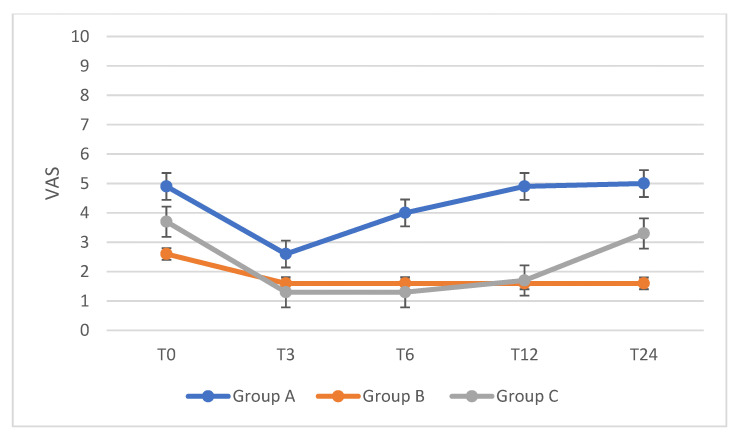
Changes of CPP VAS score over time in exclusively breastfeeding women compared to the months of breastfeeding. T0 = before pregnancy; T3 = 3-month follow-up; T6= 6-month follow-up; T12 = 12-month follow-up; T24 = 24-month follow-up. A = 1–2 months of breastfeeding; B = 3–5 months of breastfeeding; C = 6–8 months of breastfeeding.

Moreover, both duration of exclusive breastfeeding and duration of postpartum amenorrhea were significantly associated with reduced intensity of dysmenorrhea after resumed menstrual period. CPP median VAS score at T0 was 4.5, mean 4.7 ± 2. Patients in all groups reported an overall improvement, but it was statistically significant only in group C and only during the first 6 months of follow-up (median VAS 0.3 at T6, *p* = 0.002). Pain intensity increased over the time, but only subgroup A reached the VAS score reported before pregnancy. Changes over time in CPP compared to the duration of breastfeeding are shown in Figure 2. 

The prevalence of dyspareunia widely decreased in group C (50% vs. 25%) during the 24-month follow-up, but the intensity in terms of VAS score was not statistically significant. The results are reported in Table 2.

### 3.2. Effect of Breastfeeding on Ovarian Endometriomas 

All 123 patients underwent TVUS examination at the beginning of pregnancy, as well as at 6 and 24 months after childbirth. At the beginning of pregnancy, an ovarian endometrioma was found in 51/123 women (41.4%), of whom 42 (82.4%) exclusively breastfed for a period of time. In 17/51 patients (33.3%), 14 of whom exclusively breastfed, TVUS showed a reduced diameter of ovarian endometriomas (*p* = 0.041) of at least 1 cm (mean 2.71 cm ± 0.79 cm). No recurrence of disease was observed at T24. No correlation between the reduction of ovarian endometriomas and the duration of breastfeeding was found. (Table 3)

## 4. Discussion

According to the WHO, breastfeeding is one of the most effective ways to ensure child health. Exclusive breastfeeding is recommended from the first hour after birth to be continued for at least 6 months [25]. It is of paramount importance for the newborn’s unparalleled immunological and metabolic benefits, and breastfeeding may furthermore positively impact on maternal health through the hormonal feedback pathway regulation in the pituitary gland [26,39]. To our knowledge, few studies have been conducted to analyze the effect of breastfeeding on endometriosis, but some authors observed an inverse correlation between breastfeeding and the risk of developing endometriosis [37,38,41,42]. A prospective cohort study by Farland et al. investigated the association between lifetime breastfeeding, postpartum amenorrhea, and relative risk of incident endometriosis, highlighting the protective role of breastfeeding in a group of 3296 affected women [36]. Moreover, Jang et al. found that the recurrence of dysmenorrhea was significantly reduced in patients who delivered at term and breastfed [42]. Similarly, in our study there was a significant reduction in the intensity of dysmenorrhea in all women who breastfed, but the VAS pain score was lower in women providing exclusive breastfeeding compared to the intensity reported before pregnancy. This favorable effect is also confirmed by the reduced need for analgesics during the whole follow-up period. The beneficial effects of breastfeeding could be related to its action on the pituitary hormonal activity, leading to a reduction of circulating estrogens, which are specifically involved in maintenance and rise of endometriotic lesions [36]. In addition, prolonged postpartum amenorrhea may play an important role in the recurrence of endometriosis and related symptoms. In the present study, a shorter duration of amenorrhea was associated with earlier recurrence of pain symptoms. Our results are in line with the study carried out by Farland et al., who reported that the longer the amenorrhea, the lower the risk of recurrence and intensity of painful symptoms [36]. In our study, the mean time of resumed menstrual period after delivery was 6.2 months, similar to the results published by Chowdhury et al. [35], who reported that the mean lactation amenorrhea is 6 months. However, the duration of amenorrhea strongly depends on the type and the duration of breastfeeding, being longer in cases of exclusive breastfeeding. As expected, pain intensity started to increase again over time but it did not reach the intensity reported before pregnancy, particularly in patients providing prolonged exclusive breastfeeding. This was confirmed also by a reduced request for analgesics, thus showing the long-term beneficial effect of breastfeeding.

In our study breastfeeding women experienced an overall slight improvement in CPP; however, a significant decrease in pain intensity was found only in women who exclusively breastfed for at least 6–8 months. However, the small number of cases in each group, the low intensity of pain in some women before pregnancy, such as in group B, and the small number of cases of women who breastfed for more than 8 months may have influenced the results. This finding may be related to the complex physiopathology of CPP, which is, as already reported, not only related to the presence and the extent of endometriotic lesions, but also to other factors, such as adhesions and nerve involvement [2,5,6,43,44,45]. Furthermore, the high rate of comorbidities and the association with gastrointestinal disorders and autoimmune diseases may increase pelvic and abdominal pain. [46,47,48,49]. All patients reported prolonged improvement in pain symptoms, which allowed them to do without hormonal therapy and to reduce the need for analgesics, thus avoiding the possible side-effects related to treatments.

Evaluation of QoL was not a scope of our study, but it is interesting that the individual perception of psychophysical well-being improved in all women who breastfed, and particularly in those who exclusively breastfed. This effect may be related not only to pain improvement, but also to the emotional impact associated with breastfeeding [50]. 

Surprisingly, despite the reduced prevalence of pain in all patients, no statistically significant correlation between breastfeeding and dyspareunia was found, but the prevalence of painful intercourse was generally decreased, particularly in the exclusive breastfeeding group. However, it should be kept in mind that the effect of breastfeeding on sexuality and intercourse after pregnancy is difficult to analyze, as several factors may be involved. Type of delivery and hormonal changes affect the patient’s sexual life and postpartum dyspareunia may occur. According to some studies, women who experienced long-lasting perineal pain after delivery reported a negative impact on their sexual life [51,52].

Barrett et al. analyzed changes in sexual activity after pregnancy and found that perineal trauma negatively impacted postpartum dyspareunia, leading to pain during penetration and sexual intercourse for at least 3–6 months after delivery [53]. However, Leeman et al. investigated the potential role of breastfeeding on sexual function and found that the elevated amount of circulating PRL led to a lower production of ovarian androgens and estrogens, thereby decreasing sexual desire and vaginal lubrication [54]. Signorello et al. observed reduced sexual desire and occurrence of dyspareunia in healthy women after 6 months of breastfeeding (O.R. 4.4), probably due to substantial hormonal and physical changes [55]. 

Regarding the effect of breastfeeding on endometriotic lesions, at the beginning of pregnancy, TVUS revealed ovarian endometriomas in 51 women (41.4%). The size of ovarian endometriomas during the follow-up was reduced in all patients, but the decrease did not significantly correlate with the duration of breastfeeding or with the duration of amenorrhea. In addition, there was no correlation with the presence of pain symptoms. The effect of breastfeeding on endometriotic lesions seems to be difficult to assess and we cannot exclude that pregnancy itself caused a reduction in the volume of ovarian endometriomas. In fact, recently, Takami at al. reported a reduced volume of ovarian endometriomas in 68% of cases during pregnancy [56]. 

In contrast to what we expected at the beginning of the study, gestational age and type of delivery did not influence the type of breastfeeding. This could be due to the women’s strong desire to breastfeed and to the help offered by the nurses immediately after childbirth. Therefore, in this preliminary study, we did not recruit enough patients who artificially fed newborns to form a control group. The results of an ongoing prospective study including women who exclusively provide artificial milk formula may better clarify if the reduction of endometriotic lesions is related to pregnancy, to breastfeeding, or to both. The present study has some limitations: primarily, the study population was small, and a larger sample is required to confirm our data; in particular, the patients who provided complementary breastfeeding were fewer than those who exclusively breastfed. Secondly, the absence of a control group of patients who provided only artificial milk formula makes it difficult to establish how much the pregnancy itself can have influenced the results, particularly the impact on the reduction of endometriotic lesions. However, to the best of our knowledge, this is the first observational study that shows the great importance of breastfeeding in women with endometriosis, an area of research still unexplored by the scientific community. Breastfeeding is a modifiable behavior that can positively influence the course and the symptoms of endometriosis, and it may carry positive psychological and socio-economic implications [57,58,59]. Our study seems to support the positive role of breastfeeding in reducing dysmenorrhea and chronic pelvic pain in women affected by endometriosis, stabilizing the postpartum hormonal environment; this effect could be enhanced during the entire period of breastfeeding. Furthermore, it is important to emphasize the primary role of gynecologists in providing correct advice and support to women before, during, and after pregnancy. Each consultation should thus become an opportunity to inform the patients about the advantages of breastfeeding on both mother and newborn and on their relationship. In particular, prolonged breastfeeding should be recommended in women suffering from symptomatic endometriosis. Further studies on larger populations are required to confirm our data.

## 5. Conclusions

Women affected by endometriosis may benefit from exclusive and prolonged breastfeeding, which may reduce pain symptoms and prevent recurrences. In particular, our study showed that breastfeeding significantly reduces the intensity of pain symptoms. These findings correlate with the duration and intensity of breastfeeding. A reduced size of ovarian endometriomas and recurrences was also observed, although the role of breastfeeding on endometriosis lesions is still unclear. Gynecologists should therefore encourage women to start breastfeeding immediately after childbirth and to continue for a long time. The results of this study are promising, but further research with a larger sample including women with endometriosis who do not breastfeed is required to confirm the role of breastfeeding as a protective factor in women with endometriosis.

## Figures and Tables

**Table 1:** General characteristics of the study population.

**Age at delivery**	**mean ± SD**
	33.8 ± 4.7
**BMI**	**mean ± SD**
	22.2 ± 2.7
**Previous medical treatment**	***n* (%)**
	51 (41.4)
**NSAID intake**	***n* (%)**
	**109 (88.6)**
**Parity**	***n* (%)**
0	84 (68.3)
1	27 (21.9)
2	12 (9.8)
**Stage at previous surgery**	***n* (%)**
Stage III	66 (53.6)
Stage IV	57 (46.4)
**Surgical findings**	***n* (%)**
Peritoneal lesions	74 (60.1)
Adhesions	62 (50.4)
DIE	39 (31.7)
Unilateral endometriomas	76 (61.8)
Bilateral endometriomas	47 (38.2)
**Ovarian endometriomas at the beginning of pregnancy**	***n* (%)**
Unilateral	39 (76.4)
Bilateral	12 (23.6)
**Average size of ovarian endometriomas at pregnancy (cm)**	**mean ± SD**
	3.25 ± 0.91
**Prevalence of pain symptoms before pregnancy**	***n* (%)**
Dysmenorrhea	101 (82.1)
Dyspareunia	74 (60.1)
CPP	91 (73.9)
**Mean VAS score**	**mean ± SD**
Dysmenorrhea	6.1 ± 3.6
Dyspareunia	5.1 ± 2.8
CPP	4.8 ± 2
**Type of delivery**	***n* (%)**
Vaginal	60 (48.8)
Cesarean section	63 (51.2)
**Gestational age at delivery**	***n* (%)**
Preterm (<37 weeks)	18 (14.6)
Term (≥37 weeks)	105 (85.4)
**Months of postpartum amenorrhea**	**mean ± SD**
	5.6 ± 4.3

**Table 2:** Median VAS score before pregnancy and at follow-up compared to the months of breastfeeding of the 96 patients who exclusively breastfed.

**Dysmenorrhea**						
	**Group A** **(*n* = 17)**	***p*-Value**	**Group B** **(*n* = 19)**	***p*-Value**	**Group C** **(*n* = 33)**	***p*-Value**
**T0**	6.9		4.7		6.5	
T3 *	2	0.001	3.6	0.04	2.7	0.002
T6 *	4.6	0.004	3.2	0.002	2.1	<0.001
**T12**	5.4	n.s.	3.7	n.s.	2.4	<0.001
**T24**	5.4	n.s.	3.6	n.s.	4.3	<0.001
**Dyspareunia**						
	**Group A** **(*n* = 9)**	***p*-Value**	**Group B** **(*n* = 22)**	***p*-Value**	**Group C** **(*n* = 15)**	***p*-Value**
**T0**	5.4	n.s.	5.5	n.s.	5.4	n.s.
**T3**	4.9	n.s.	5.2	n.s.	5.1	n.s.
**T6**	4.8	n.s.	4.7	n.s.	4.6	n.s.
**T12**	5.3	n.s.	4.9	n.s.	4.4	n.s.
**T24**	5.2	n.s.	5.1	n.s.	4.7	n.s.
**CPP**						
	**Group A** **(*n* = 19)**	***p*-Value**	**Group B** **(*n* = 25)**	***p*-Value**	**Group C** **(*n* = 23)**	***p*-Value**
**T0**	4.9		2.6		3.7	
**T3**	2.6	0.05	1.6	n.s.	1.3	0.002
**T6**	4.0	n.s.	1.6	n.s.	1.3	0.002
**T12**	4.9	n.s.	1.6	n.s.	1.7	0.04
**T24**	5.0	n.s.	1.6	n.s.	3.3	n.s.

T0 = before pregnancy; T3 = 3-month follow-up; T6 = 6-month follow-up; T12 = 12-month follow-up; T24 = 24-month follow-up. A = 1–2 months of breastfeeding; B = 3–5 months of breastfeeding; C = 6–8 months of breastfeeding. n.s. = not significant. * VAS calculated only in patients with early resumed menstrual period.

**Table 3:** Size of ovarian endometriomas before pregnancy and at 6- and 24-month follow-up periods.

	All Patients (*n* = 51) (mean cm ± SD)	Exclusive Breastfeeding Patients (*n* = 42) (mean cm ± SD)	*p*-Value
**Before pregnancy**	3.25 ± 0.91	3.23 ± 0.91	
**6-month follow-up**	3.01 ± 0.81	2.98 ± 0.79	0.041
**24-month follow-up**	2.71 ± 0.79	2.69 ± 0.76	

## Data Availability

The data presented in this study are not publicly available due to privacy policy but are available on request from the corresponding author.
